# Brazilian Green Propolis Suppresses the Hypoxia-Induced Neuroinflammatory Responses by Inhibiting NF-**κ**B Activation in Microglia

**DOI:** 10.1155/2013/906726

**Published:** 2013-08-01

**Authors:** Zhou Wu, Aiqin Zhu, Fumiko Takayama, Ryo Okada, Yicong Liu, Yuka Harada, Shizheng Wu, Hiroshi Nakanishi

**Affiliations:** ^1^Department of Aging Science and Pharmacology, Faculty of Dental Science, Kyushu University, Fukuoka 812-8582, Japan; ^2^Institution of Geriatric Qinghai Provincial Hospital, Xining 810007, China

## Abstract

Hypoxia has been recently proposed as a neuroinflammatogen, which drives microglia to produce proinflammatory cytokines, including interleukin-1**β** (IL-1**β**), tumor necrosis factor-**α** (TNF-**α**), and IL-6. Considering the fact that propolis has hepatoprotective, antitumor, antioxidative, and anti-inflammatory effects, propolis may have protective effects against the hypoxia-induced neuroinflammatory responses. In this study, propolis (50 **μ**g/mL) was found to significantly inhibit the hypoxia-induced cytotoxicity and the release of proinflammatory cytokines, including IL-1**β**, TNF-**α**, and IL-6, by MG6 microglia following hypoxic exposure (1% O_2_, 24 h). Furthermore, propolis significantly inhibited the hypoxia-induced generation of reactive oxygen species (ROS) from mitochondria and the activation of nuclear factor-**κ**B (NF-**κ**B) in microglia. Moreover, systemic treatment with propolis (8.33 mg/kg, 2 times/day, i.p.) for 7 days significantly suppressed the microglial expression of IL-1**β**, TNF-**α**, IL-6, and 8-oxo-deoxyguanosine, a biomarker for oxidative damaged DNA, in the somatosensory cortex of mice subjected to hypoxia exposure (10% O_2_, 4 h). These observations indicate that propolis suppresses the hypoxia-induced neuroinflammatory responses through inhibition of the NF-**κ**B activation in microglia. Furthermore, increased generation of ROS from the mitochondria is responsible for the NF-**κ**B activation. Therefore, propolis may be beneficial in preventing hypoxia-induced neuroinflammation.

## 1. Introduction

The brain is highly susceptible to being damaged by hypoxia because of its high demand for oxygen supply [1]. Microglia are resident innate immune cells in the brain, constituting the first line of defense against brain insults [2, 3]. It is generally accepted that hypoxia is one of the neuroinflammatogens in the brain, because hypoxia activates microglia to provoke excessive secretion of proinflammatory cytokines, including interleukin-1*β* (IL-1*β*) and tumor necrosis factor-*α* (TNF-*α*) [4–7]. It is also known that proinflammatory cytokines secreted by microglia promote cognitive deficits in aged people and Alzheimer's disease (AD) patients [8, 9]. In our previous studies, we have found that enhanced production of reactive oxygen species (ROS) due to the increased mitochondrial DNA damage in microglia is responsible for exaggerated inflammatory responses in aged animals after treatment with lipopolysaccharide, because the increased intracellular ROS level activates nuclear factor-*κ*B (NF-*κ*B) which regulates the expression of several proinflammatory cytokines [10]. Hypoxia can drive microglia to generate ROS [11–14]. Therefore, it is reasonable to consider that hypoxia activates NF-*κ*B to induce exaggerated inflammatory response by microglia through enhanced production of ROS due to the mitochondrial DNA damage.

Propolis is a resinous substance produced by honeybees as a defense against intruders. It has relevant therapeutic properties that have been used since ancient times. The chemical composition of propolis depends on the local floral at the site of collection [15, 16]. Considering the fact that propolis has hepatoprotective, antitumor, antioxidative, and anti-inflammatory effects [17–20], propolis may have protective effects against the hypoxia-induced neuroinflammatory responses. In this study, we provide the first evidence that propolis can significantly inhibits the secretion of IL-1*β*, TNF-*α*, and interleukin-6 (IL-6) by microglia through inhibition of the NF-*κ*B activation in microglia. Furthermore, propolis significantly inhibit the increased generation of ROS from the mitochondria that is responsible for the NF-*κ*B activation. These observations suggest that propolis may be useful to prevent hypoxia-induced neuroinflammation.

## 2. Material and Methods

### 2.1. Reagents

The Brazilian green propolis ethanol extract (propolis) was purchased from Yamada Apiculture Center, Inc Ltd. (Okayama, Japan). The suitable concentration of ethanol for cell culture was titrated in order to prevent the interference induced by the ethanol solvent. Goat anti-mature IL-1*β* (mIL-1*β*), goat anti-TNF-*α*, goat anti-IL-6, and mouse anti-phospho-I*κ*B*α*, rabbit anti-I*κ*B*α*, mouse anti-phospho-p65 were purchased from Santa Cruz Biotechnology (Delaware Avenue Santa Cruz, CA). Rabbit polyclonal anti-Iba1 antibody was purchased from Wako Pure Chemicals (Sapporo, Japan), and mouse monoclonal anti-8-oxo-deoxyguanosine (8-oxo-dG) was purchased from NOF Corporation (Kyoto, Japan).

### 2.2. Microglial Cell Culture

The c-myc-immortalized mouse microglial cell line, MG6 (RIKEN Cell Bank, Tsukuba, Japan), was maintained in Dulbecco's modified Eagle's medium containing 10% fetal bovine serum (ICN Biomedicals, Inc.) supplemented with 100 *μ*M of *β*-mercaptoethanol, 10 *μ*g/mL of insulin, 100 *μ*g/mL of streptomycin, and 100 U/mL of penicillin (BD Falcon, Franklin Lakes, NJ) [21, 22]. 

### 2.3. Assay for Cell Survival

Relative cell viability was measured using CellQuanti-MTT Cell Viability Assay Kits (BioAssay Systems, Hayward, USA). The assay was performed according to the protocol provided by manufacturer. The absorbency at 570 nm was performed by using a microplate reader. 

### 2.4. Hypoxic Exposure

For *in vitro* studies, MG6 microglia (cell density of 2 × 10^4^  cells/mL) were plated overnight and then cultivated under the normoxia (20% O_2_, 5% CO_2_) or hypoxia (1% O_2_, 5% CO_2_, and 92% N_2_) at 37°C for the indicated periods using a chamber (Model: MCO 18M; Sanyo Biomedical Electrical Co., Ltd., Tokyo, Japan). For* in vivo* studies, eighteen C57B/6N mice (4-week old) with or without treatment of propolis (9 mice each, 8.33 mg/kg, 2 times/day, i.p.) for 7 days were exposed to hypoxia by placing in a chamber (Model: MCO 18 M) filled with a gas mixture of 10% oxygen and 95% nitrogen for 4 h. The mice were then allowed to recover under normoxic conditions for 24 h before killing. Another group of eighteen mice kept outside the chamber were used as matched controls. This study was approved by the Institutional Animal Care and Use Committee of Kyushu University.

### 2.5. Tissue Preparation

Mice were exposed to normoxia or hypoxia with pretreatment of propoplis (8.33 mg/kg, 2 times/day). Mice were exposed to hypoxia with pretreatment of 0.01 M phosphate-buffered saline (PBS, pH 7.4, 2 times/day) as control. The mice were anesthetized with sodium pentobarbital (30 mg/kg, i.p.) and then were perfused intracardially with PBS (pH 7.4) and periodate lysine paraformaldehyde (PLP) fixative containing 0.01 M sodium metaperiodate, 0.075 M l-lysine-HCl, 2% paraformaldehyde, and 0.03% phosphate buffer (pH 6.2). The brains were removed and immersed in the same fixative for 6 h at 4°C. The specimens were cryoprotected for 2 days in 30% sucrose in PBS and then were embedded in an optimal cutting temperature compound (Sakura Finetechnical Co., Ltd., Tokyo, Japan). Serial coronal frozen sections (14 *μ*m) of the somatosensory cortex for staining with immunohistochemistry and double-immunofluorescent staining were prepared as previously reported [23, 24].

### 2.6. Detection of Mitochondrial ROS

Mitochondrial ROS was measured using MitoSOX Red (Invitrogen, USA), which is a live-cell permeant and is rapidly and selectively targeted to mitochondria [25]. Once in the mitochondria, MitoSOX Red reagent is oxidized by superoxide and exhibits red fluorescence (with excitation at 510 nm and emission at 580 nm). The cultured MG6 microglia (cell density of 1 × 10^5^ cells/mL) were exposed to nomoxia or hypoxia in the presence or absence of propolis (50 *μ*g/mL). The cells were collected at 24 h after treatment and then incubated in Hank's balanced salt solution (HBSS) containing 5 mM MitoSOX Red for 10 min at 37°C. After incubation, the cells were washed with PBS twice the cells were then mounted in a warm buffer for imaging. Images were collected using a ×20 objective lens (NA = 0.50, 200x magnification, yielding a frame of 0.575 mm^2^). The procedure resulted in arbitrary optical density values on a scale of 0 (background staining) to 255.

### 2.7. Immunofluorescence Imaging

MG6 microglia exposed to normoxia or hypoxia for 60 min using a chamber (Model: MCO 18 M) in the presence or absence of propolis (50 *μ*g/mL) were fixed with 4% paraformaldehyde and then incubated with the mouse anti-NF-*κ*B p65 (1 : 500, F-6) overnight at 4°C. After incubated with anti-mouse Alexa 488 (1 : 2000, Jackson Immunoresearch Lab. Inc.) at 4°C for 2 h, they were then incubated with Hoechst (1 : 2000, Sigma-Aldrich, Japan) and mounted in the antifading medium Vectashield. Fluorescence images were taken using a confocal laser scanning microscope (CLSM; C2si, Nikon, Japan).

The sections were hydrated and treated with 10% donkey serum for 1 h at 25°C and then were incubated with each primary antibody overnight at 4°C. The primary antibodies were goat polyclonal anti-IL-1*β* antibody (1 : 500), goat polyclonal anti-TNF-*α* (1 : 500), goat polyclonal anti-IL-6 (1 : 500), and mouse monoclonal anti-8-oxo-dG (1 : 500) antibodies mixed with rabbit polyclonal anti-Iba1 antibody (1 : 5000). The sections were washed with PBS and incubated with a mixture of FITC-conjugated and rhodamine-conjugated secondary antibodies for 2 h at 25°C. The sections were washed, mounted in the antifading medium Vectashield (Vector Laboratory), and then were examined by a confocal laser scanning microscope (CLSM, C2si, Nikon, Japan). CLSM images of individual sections were taken as a stack at 1 *μ*m step size along the *z*-direction with a 20x objective (Numerical Aperture = 0.5), zoom factor 1.0. A rectangle (1024 × 1024 pixels) corresponding to the size of 450 × 450 *μ*m was used as the counting frame. CLSM images were shown as the middle of the stacked images.

### 2.8. Enzyme-Linked Immunosorbant Assay (ELISA)

The cultured MG6 microglia (density of 5 × 10^5^ cells/mL) with propolis (50 *μ*g/mL) were subjected to hypoxia in a chamber filled with a mixture of gases containing 1% O_2_, 5% CO_2_, and 92% nitrogen at 37°C. MG6 microglia were incubated in 21% O_2_, and 5% CO_2_ at as 37°C as the normoxic control. The condition medium was collected at 6, 12, 24, and 48 hours after the above incubation, and the amounts of IL-1*β*, TNF-*α*, and IL-6 released from microglia were measured using the enzyme-linked immunosorbent assay (ELISA) kits (R&D Systems) following the protocol provided by the manufacturer. The absorbency at 450 nm was measured using a microplate reader.

### 2.9. Electrophoresis and Immunoblotting

MG6 microglia were cultured at a density of 5 × 10^5^ cells/mL, and the cytosolic samples were collected at the time points 15, 30, and 60 min after hypoxic exposed hypoxia (1% O_2_). The samples were electrophoresed in 15% or 12% SDS-polyacrylamide gels, and the proteins on SDS gels were transferred electrophoretically to nitrocellulose membranes. Following blocking, the membranes were incubated at 4°C overnight under gentle agitation with each primary antibody: mouse anti-phosphorylated I*κ*B*α* (1 : 1000) and rabbit anti-I*κ*B*α* (1 : 1000) antibodies overnight at 4°C. After washing, the membranes were incubated with horseradish-peroxidase- (HRP-) labeled anti-mouse (1 : 2000, Beckman Coulter) and anti-rabbit (1 : 2000, Beckman Coulter) antibodies for 2 hours at 24°C and then detected using an enhanced chemiluminescence detection system (ECK kit, Amersham Pharmacia Biotech) with an image analyzer (LAS-4010, GE health care, Uppsala, Sweden).

### 2.10. Statistical Analysis

The data are represented as the means ± SEM. The statistical analyses were performed using a one-way or two-way analysis of variance (ANOVA) with a post hoc Tukey's test using the GraphPad Prism software package. A value of *P* < 0.05 was considered to indicate statistical significance (GraphPad Software Inc., San Diego, CA, USA). 

## 3. Results

### 3.1. Effects of Propolis on the Hypoxia-Induced Reduction of Microglia Viability and Hypoxia-Induced Mitochondria-Derived ROS by Microglia

**Figure 1:**
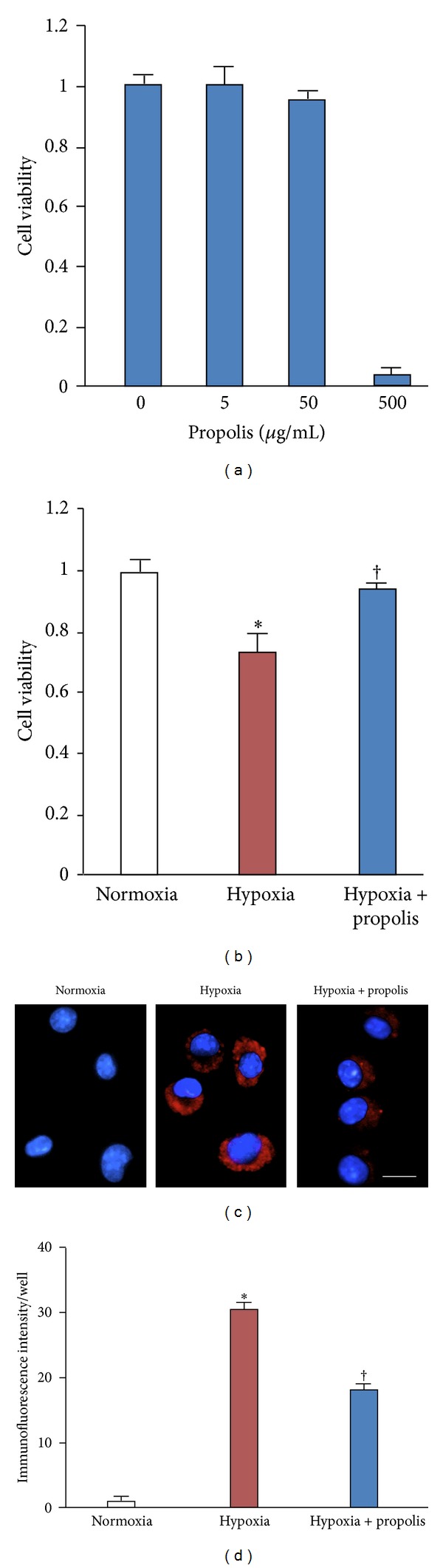
Effect of propolis on hypoxia-induced mitochondria ROS associated cytotoxicity in cultured microglia. (a) Cell viability in MG6 microglia in the presence of propolis with different three concentrations. (b) Cell viability of MG6 microglia exposed to normoxia (20% O_2_) or hypoxia (1% O_2_) in the presence or absence of propolis (50 *μ*g/mL) for 24 h. Each column and bar represent mean ± SEM (*n* = 4 each). An asterisk indicates a statistically significant difference from the value in Normoxia (*P < 0.05). A sword indicates a statistically significant difference from the value in hypoxia (^†^ *P* < 0.05). (c) Fluorescent mages of MitoSOX Red fluorescence signals in MG6 microglia exposed to normoxia (20% O_2_) or hypoxia (1% O_2_) in the presence or absence of propolis (50 *μ*g/mL) for 24 h. Scale bar = 10 *μ*m. (d) The quantitative analyses of MitoSOX Red fluorescence signal intensity in (c). Each column and bar represent the mean ± SEM (*n* = 4 each). An asterisk indicates a statistically significant difference from the value in normoxia (*P < 0.05). A sword indicates a statistically significant difference from the value in hypoxia (^†^ *P* < 0.05).

We first investigated the effects of propolis on the cell viability of MG6 microglia using MTT assay. The mean cell viability was not significantly changed after treatment with propolis with the final concentrations of 5 or 50 *μ*g/mL (Figure 1(a)). On the other hand, the mean cell viability was significantly reduced after treatment with propolis with the final concentration of 500 mg/mL. Therefore, we used propolis with the concentration of 50 *μ*g/mL to examine its effects on hypoxia-induced reduction of microglial cell viability. As shown in Figure 1(b), hypoxic exposure (1% O_2_, 24 h) significantly reduced the mean cell viability of MG6 microglia. Treatment with propolis (50 *μ*g/mL) significantly restored the hypoxia-induced reduction of microglial cell viability (Figure 1(b)). 

Hypoxia drives microglia to generate ROS. In our previous study, the mitochondria in microglial were found to be most susceptible to oxidative damage [10, 26, 27]. These facts prompted us to examine hypoxia-induced mitochondrial oxidant generation in microglia using oxidation of the MitoSOX Red probe, a mitochondrially targeted hydroethidine derivative [25]. The mean immunofluorescence intensity of MitoSOX Red oxidation was significantly increased in MG6 microglia at 24 h after hypoxia (Figures 1(c) and 1(d)). Propolis (50 *μ*g/mL) significantly inhibited the hypoxia-induced increase in the mean fluorescent intensity of MitoSOX Red probe in microglia (Figures 1(c) and 1(d)). These results demonstrate that propolis inhibits the hypoxia-induced ROS generation from mitochondria in microglia.

### 3.2. Effects of Propolis on the Hypoxia-Induced Secretion of Proinflammatory Cytokines by Cultured Microglia

**Figure 2:**
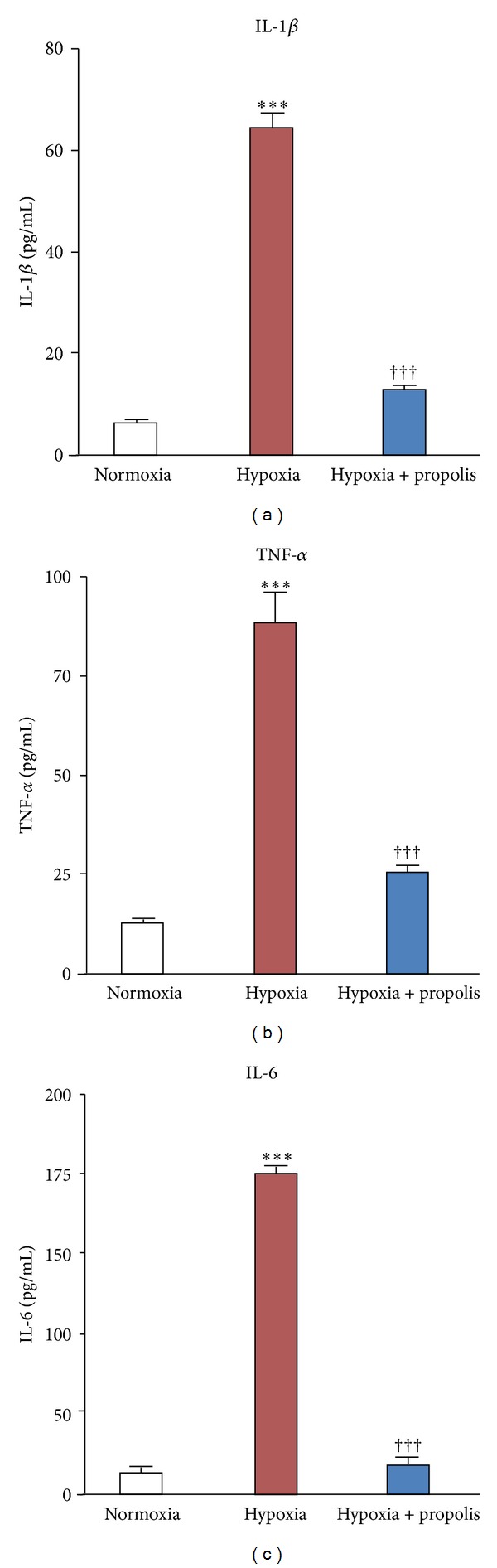
Inhibitory effects of propolis on the hypoxia-induced proinflammatory cytokine secretion by cultured microglia. The mean concentration of IL-1*β*, TNF-*α*, and IL-6 in the culture medium of MG6 microglia exposed to normoxia (20% O_2_) or hypoxia (1% O_2_) in the presence or absence of propolis (50 *μ*g/mL) for 24 h was measured by ELISA. Each column and bar represent the mean ± SEM (*n* = 4 each). Asterisks indicate a statistically significant difference from the value in normoxia (***P < 0.001). Swords indicate a statistically significant difference from the value in hypoxia (^†††^ *P* < 0.001).

Next, the effects of propolis on the hypoxia-induced secretion of proinflammatory cytokines by microglia were examined. IL-1*β*, TNF-*α*, and IL-6 secreted by MG6 microglia into the culture medium were measure by ELISA after hypoxic exposure (1% O_2_, 24 h). The mean concentrations of IL-1*β*, TNF-*α*, and IL-6 in the culture medium of microglia by microglial cells were significantly increased at 24 h after exposure to hypoxia (Figure 2). Propolis (50 *μ*g/mL) significantly inhibited the hypoxia-induced secretion of IL-1*β*, TNF-*α*, and IL-6 by microglia (Figure 2). 

### 3.3. Effects of Propolis on Hypoxia-Induced Activation of NF-*κ*B by Microglia

**Figure 3:**
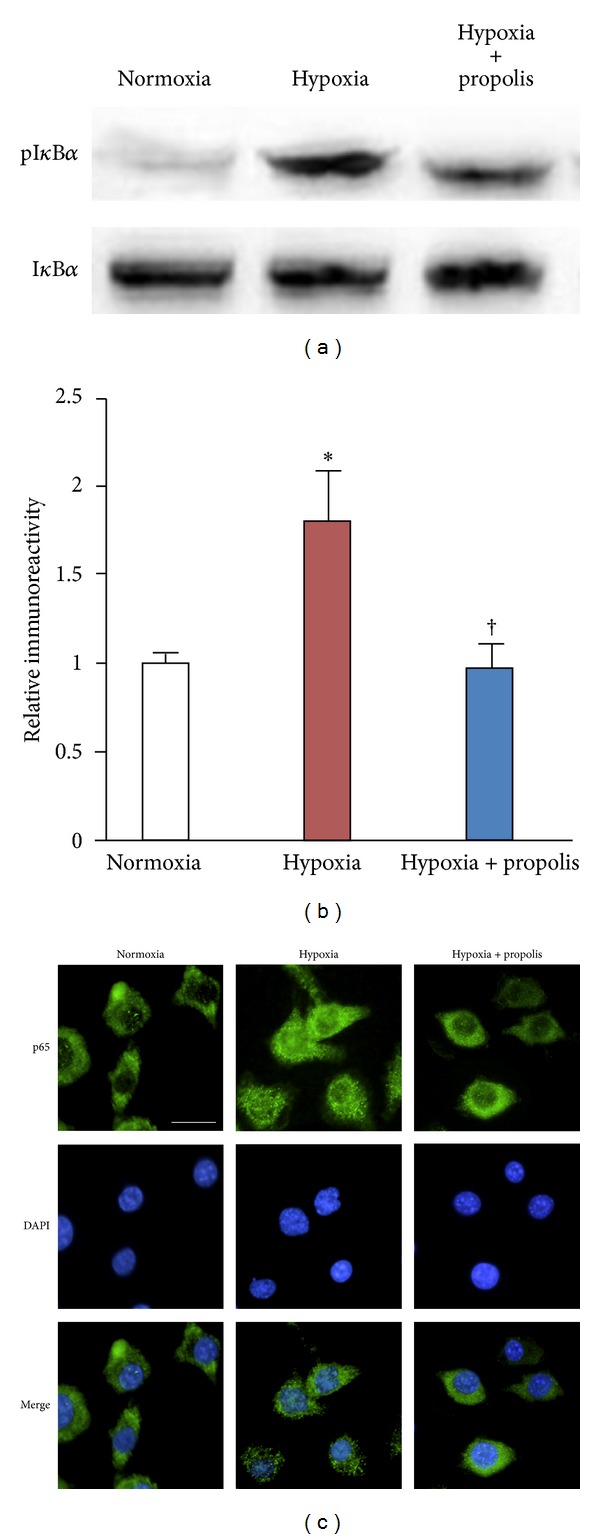
Inhibitory effects of propolis on the hypoxia-induced NF-*κ*B activation in cultured microglia. (a) Phosphorylation of I*κ*B*α* in MG6 microglia exposed to normoxia (20% O_2_) or hypoxia (1% O_2_) in the presence or absence of propolis (50 *μ*g/mL) for 24 h. (b) The quantitative analyses of immunoblots in (a). Each column and bar represent the mean ± SEM (*n* = 4 each). An asterisk indicates a statistically significant difference from the value in Normoxia (*P < 0.05). A sword indicates a statistically significant difference from the value in hypoxia (^†^ *P* < 0.05). (c) Immunofluorescent CLMS images of p65 (green) with Hoechst-stained nuclei (blue) in MG6 microglia exposed to normoxia (20% O_2_) or hypoxia (1% O_2_) in the presence or absence of propolis (50 *μ*g/mL) for 24 h.

The effects of propolis on the NF-*κ*B activation after exposure to hypoxia were next examined, because NF-*κ*B regulates the expression of several proinflammatory cytokines, including IL-1*β*, TNF-*α*, and IL-6. The expression of I*κ*B*α* phosphorylation in MG6 microglia was significantly increased after hypoxia (Figures 3(a) and 3(b)). Propolis (50 *μ*g/mL) significantly inhibited the hypoxia-induced phosphorylation of I*κ*B*α* in microglia (Figures 3(a) and 3(b)). Furthermore, the nuclear translocation of p65 was induced in MG6 microglia at 60 min after hypoxia (Figure 3(c)). Propolis (50 *μ*g/mL) markedly inhibited the hypoxia-induced nuclear translocation of p65 in microglia (Figure 3(c)). These results demonstrate that propolis suppresses the hypoxia-induced neuroinflammatory responses by inhibiting NF-*κ*B activation in microglia.

### 3.4. Effects of Propolis on the Neuroinflammatory Responses in the Somatosensory Cortex of Mice Exposed to Hypoxia

**Figure 4:**
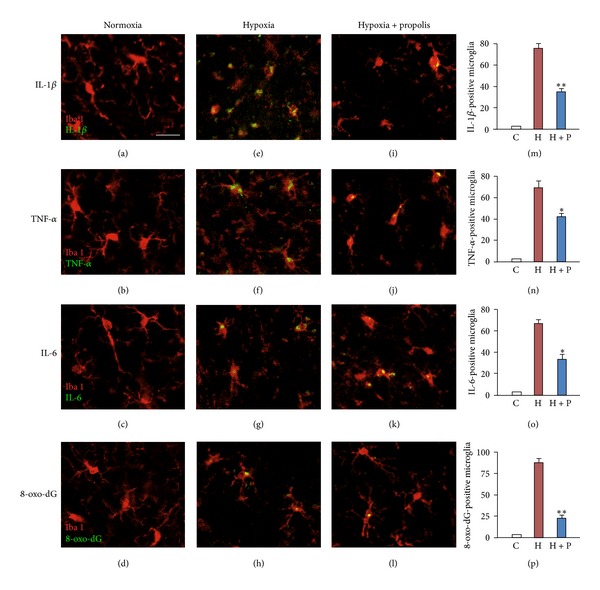
Inhibitory effects of propolis on the expression of IL-1*β*, TNF-*α*, IL-6, and 8-oxo-dG in the cortical microglia of mice exposed to hypoxia. Immunofluorescent CLMS images of IL-1*β* (a, e, i), TNF-*α* (b, f, j), IL-6 (c, g, k), and 8-oxo-dG (d, h, l) in the Iba1-positve cortical microglia of mice exposed to normoxia (20% O_2_) or hypoxia (10% O_2_) for 4 h with or without pretreatment of propolis (8.33 mg/kg, 2 times/day). (m–p) The mean cell number of IL-1*β*-positive (m), TNF-*α*-positive (n), IL-6-positive (o) and 8-oxo-dG-positive (p) Iba1-positive microglia in the somatosensory cortex per 0.15 mm^2^. Each column and bar represent the mean ± SEM (*n* = 3 each). Asterisks indicate a statistically significant difference from the value in normoxia (*P < 0.05, **P < 0.01). Scale bar = 20 *μ*m.

Finally, the effects of propolis on the cortical microglia in mice exposed to hypoxia were exposed. Under the normoxic condition, the cortical microglia exhibited ramified morphology (Figures 4(a)–4(d)). In contrast, the cortical microglia showed hyperactivated morphology, which was characterized by enlarged cell bodies with short processes at 4 h after hypoxia which recover under normoxic conditions for 24 h (Figures 4(e)–4(h)). Furthermore, the mean cell numbers of cortical microglia positive for immunoreactivities of IL-1*β*, TNF-*α*, IL-6, and 8-oxo-dG were significantly increased in mice at 4 h after hypoxia (Figures 4(e)–4(h), and 4(m)–4(p)). When mice were chronically treated with propolis (8.33 mg/kg, 2 times/day, i.p.) for 7 days before exposure to hypoxia, the morphology of the cortical microglia was characterized by small cell bodies with long processes, similar to the ramified microglia under the normoxic condition (Figures 4(i)–4(l)). At the same time, the mean cell numbers of cortical microglia positive for the immunoreactivities of IL-1*β*, TNF-*α*, IL-6, and 8-oxo-dG were significantly reduced (Figures 4(i)–4(l), and 4(m)–4(p)). However, the pretreatment of PBS (controls) did not show inhibition of the activated morphology and the immunoreactivities of IL-1*β*, TNF-*α*, IL-6, and 8-oxo-dG in microglia of hypoxia exposed mice (data not shown).

## 4. Discussion

The major finding of this study is that propolis significantly inhibits the hypoxia-induced activation of NF-*κ*B-dependent neuroinflammatory pathway in microglia. NF-*κ*B is a transcription factor that encodes genes of the proinflammatory cytokines, including IL-1*β*, TNF-*α* and IL-6 [28]. It is also known that NF-*κ*B activation is facilitated by conditions associated with an increased intracellular redox state [29]. In the present study, the mean fluorescent intensity of MitoSOX Red probe, a marker for mitochondria-derived ROS generation, was found to be significantly increased in microglia following hypoxia. Furthermore, the increased expression of 8-oxo-dG, a biomarker for oxidative damaged DNA [30], was observed mainly in the cytosol of microglia after hypoxia, suggesting the mitochondrial origin of damaged DNA. We have previously found that ROS damages the mitochondrial DNA and the damaged mitochondria DNA, in turn, impairs the respiratory chain, forming a vicious cycle to promote the ROS generation [10]. Taken together, the mitochondrial DNA damage after hypoxia is considered to be a major causative factor for the increased ROS production, which activates the NF-*κ*B-dependent neuroinflammatory pathway. Therefore, propolis may protect the mitochondrial DNA against hypoxia-induced oxidative stress dependently on its antioxidant properties [18, 19, 31].

Stroke is the most common form of hypoxia-ischemic brain injury and remains a major challenge to public health due to its high incidence and life-threatening nature [32]. In the Western world, over 70% of individuals experiencing a stroke are over 65 years of age. Since life expectancy continues to grow, the absolute number of individuals with stroke will further increase in the future [33]. Oxidative stress and neuroninflammation are known as the two important pathophysiological mechanisms involved during hypoxia-ischemic brain injury [33], because mice lacking the p50 subunit of NF-*κ*B develop significantly smaller infarcts after transient focal ischemia [34] and antioxidants reduce infarct volume and improve behavior deficits [35]. More evidence further explores the critical role of microglia in stroke progression, because the experimental and postmortem studies reveal the presence of activated microglia in the brain of stroke patients [36, 37] and microglia are clarified as the major cell population that leads to NF-*κ*B-dependent upregulation of proinflammatory molecules, such as TNF-*α* and IL-1*β* during stroke. Therefore, propolis efficiently attenuates the hypoxia-induced activation of NF-*κ*B-dependent neuroinflammatory pathway in microglia and may be beneficial in the prevention and treatment of stroke, because blockade of microglia activation prevents hypoxia-ischemic brain injury [38]. 

Recently, much attention has been paid to the association of hypoxia with cognitive deficits. It is well known that hypoxia suffering mountaineers have demonstrably poorer memory and concentration, and the effect of hypoxia is sustained for significant periods of time after returning from altitude [39, 40]. In addition, we have previously found that healthy individuals who are living in high altitude in excursions are with cognitive defects [41]. A similar decline in memory arising from brief hypoxic exposure has been reported in experimental animals [42]. Brain oxygen levels are largely dependent on cerebral blood flow [43, 44], which declines with aging [44]. Furthermore, the cerebral blood flow was 20% lower in AD than in the age-matched nondemented control group [45]. Older adults with low cerebral oxygen levels showed more cognitive dysfunction than those with normal levels [46]. Therefore, the chronic hypoxia may contribute to the cognitive decline in aging and aging-related neurodegenerative diseases, such as AD [47, 48]. Recently, we have reported that the exaggerated neuroinflammatory responses evoked by microglia are associated with an impairment of the hippocampal long-term potentiation (LTP), a cellular basis for memory and learning, because minocycline, a known inhibitor of microglial activation, significantly improved systemic inflammation-induced impairment of LTP in the middle-aged animals [49]. Therefore, it is reasonable to consider that propolis efficiently attenuates hypoxia-induced NF-*κ*B-dependent neuroinflammatory pathway in microglia and may be beneficial in preventing of neurodegenerative diseases-related cognitive deficits.

People living in high altitudes are daily exposed to hypoxia. Our preliminary human experiments in the high altitudes show that the mean level of proinflammatory mediators in the blood of a propolis-treated elderly group is significantly lower than that of a nontreated group. Furthermore, the propolis-treated elderly group shows significantly higher scores of cognitive tests than the nontreated elderly group (unpublished data), which suggests that propolis may also helpful to prevent the aging-related cognitive deficits.

## 5. Conclusion

The present study provides the first evidence of potential protective effects of propolis on the hypoxia-induced neuroinflammatory responses. The protective effects may involve a reduction in oxidative stress and NF-*κ*B-dependent pathway in microglia. Thus making it beneficial in the prevention and treatment of hypoxia/ischemia-induced cognitive deficits. Our ongoing investigation is to clarify the synergistic and additive effects of individual propolis components in anti-neuroinflammation.
